# Decreased treatment of acne among adolescents in skin of color populations: An examination through the perspective of the National College Health Assessment

**DOI:** 10.1016/j.jdin.2024.01.003

**Published:** 2024-01-12

**Authors:** Sarah J. Shareef, Nader Rayyan, Alexander Woods, Kurt Ashack

**Affiliations:** a Michigan State University College of Human Medicine, East Lansing, Michigan; b Department of Dermatology, University of Illinois-Chicago College of Medicine, Chicago, Illinois; c Dermatology Associates of West Michigan, Grand Rapids, Michigan

**Keywords:** acne, adolescent health, pediatric dermatology, skin of color

*To the Editor:* Acne is an inflammatory skin condition initially affecting adolescent populations with an overall favorable prognosis.^1^^,^^2^ However, acne may result in permanent facial scarring and has been found to correlate with anxiety, depression, decreased self-esteem, and social withdrawal.^2^ In addition, the duration of acne correlates with a negative quality of life and may have a long-term impact on psychological well-being.^3^^,^^4^ With the continuation of acne into early adulthood, understanding treatment utilization among patients is important to identify where to further support initiatives to prevent disease sequelae. The American College Health Association administers the National College Health Assessment (NCHA) to assess undergraduate and graduate student health, including questions focused on acne.^5^ Our objective was to assess acne care among young-adults of different demographics, especially of skin of color populations.

Following data request from the NCHA, 195,965 responses surveyed by the American College Health Association-NCHA from 2019 to 2021 were included for analysis for acne trends (Table I). Incomplete questionnaires for the isolated questions were excluded from analysis. Results of the questionnaire were stratified by demographics, and descriptive statistics were conducted to compare results (Table II).

**Table I:** Questions analyzed by the National College Health Assessment (version III) from Fall 2019 to Spring 2021 by undergraduate and graduate respondents from institutions partnered with the American College Health Association

Questions related to acne and demographics by the ACHA-NCHA (version III) Fall 2019-Spring 2021		
Question code	Question	Result option analyzed
N3Q75A	How do you usually describe yourself?	American Indian or Native Alaskan
		Asian or Asian American
		Black or African American (B/AA)
		Hispanic or Latino/a/x
		Middle Eastern/North African (MENA) or Arab origin
		Native Hawaiian or other Pacific Islander Native
		White
		Biracial or multiracial
N3Q65A	Have you ever been diagnosed by a health care or mental health professional with any of the following ongoing or chronic conditions?	Acne
N3Q65T	Have you had an appointment and/or discussion with a health care or mental health professional for the following condition within the last 12 mo?	Acne
N3Q65W	Have you received treatment for the following condition by a health care or mental health professional within the last 12 mo?	Acne

*ACHA*, American College Health Association; *NCHA*, National College Health Assessment.

**Table II:** Data distribution by self-identified race/ethnicity of those that received a diagnosis of acne, sought care in the past 12 months, and received treatment for their acne

Race/ethnicity	Number of respondents (*n*)	Ever received an acne diagnosis *n* (%)	Of those that received a diagnosis, those that sought care in the past 12 months *n* (%)	Of those that received a diagnosis, those that received treatment in the past 12 months *n* (%)
American Indian or Native Alaskan	4119	1034 (25.1%)	412 (39.8%)	347 (33.5%)
Asian or Asian American	32,472	7475 (23%)	2804 (37.5%)	2521 (33.7%)
Black or African American	12,351	2525 (20.4%)	1035 (40.1%)	863 (34.2%)
Hispanic or Latino/a/x	31,686	7351 (23.2%)	2909 (39.5%)	2512 (34.1%)
Middle Eastern/North African or Arab origin	3501	970 (27.7%)	394 (40.6%)	335 (34.5%)
Native Hawaiian or other Pacific Islander Native	1278	322 (25.2%)	120 (37.3%)	101 (31.4%)
White	125,068	37,875 (30.2%)	14,962 (39.5%)	13,224 (47.4%)
Biracial or multiracial	9510	2685 (28.2%)	978 (36.4%)	851 (31.7%)

Of the respondents, 53,788 (27.4%) indicated they had ever been diagnosed with acne by a health care professional. Of those diagnosed with acne, 21,119 (39.4%) of respondents sought care for their acne in the past 12 months; of those that sought care in the past year, 18,583 (87.9%) of these respondents received treatment for their acne.

Of note, those identifying as white had the highest proportion of their population to ever being diagnosed with acne (30.2%), while Black or African American respondents had the lowest rates (20.4%) (Table II). Interestingly, Middle Eastern/North African and Black/African American respondents had the highest rates for their population to seek care after receiving a diagnosis (40.6% and 40.1%, respectively). White respondents had a >10% higher treatment rate in the past year compared to other non-White respondents, with Native Hawaiian/Pacific Islander and biracial/multiracial groups having the lowest rates (Table II).

The high rates of initial diagnosis, but low rate of treatment follow-up for acne among non-White populations may be attributed to access to dermatologic care, patient knowledge of available treatment, cost of treatments, or physician knowledge of treating acne in these populations.^1^ Limitations to this study include the differing number of respondents among each demographic and to include those who may have acne but have not been diagnosed by a physician or active acne at the time of surveillance.

Despite this, data from the NCHA demonstrated a unique perspective into the dermatologic needs of young-adults. This further highlights the need in the field of dermatology to increase support for patients of diverse backgrounds, and increase access to treatment for acne, to decrease the negative physical and psychosocial impact of the condition.^2^ This may be accomplished by creating a bridge in care by pediatricians to family medicine practitioners to facilitate the referral to dermatologists. In addition, targeted patient outreach and education programs may further bridge the gaps in health conditions impacting the health of skin of color populations.

## Conflicts of interest

None disclosed.
